# Antihypertensive and antioxidant effects of dietary black sesame meal in pre-hypertensive humans

**DOI:** 10.1186/1475-2891-10-82

**Published:** 2011-08-09

**Authors:** Jatuporn Wichitsranoi, Natthida Weerapreeyakul, Patcharee Boonsiri, Chatri Settasatian, Nongnuch Settasatian, Nantarat Komanasin, Suchart Sirijaichingkul, Yaovalak Teerajetgul, Nuchanart Rangkadilok, Naruemon Leelayuwat

**Affiliations:** 1 Faculty of Physical Therapy, Mahidol University, Nakhon Pathom 73170, Thailand; 2 Faculty of Pharmaceutical Sciences, Khon Kaen University, Khon Kaen 40002, Thailand; 3 Department of Biochemistry, Faculty of Medicine, Khon Kaen University, Khon Kaen 40002, Thailand; 4 Department of Pathology, Faculty of Medicine, Khon Kaen University, Khon Kaen 40002, Thailand; 5 Department of Clinical Chemistry, Faculty of Associated Medical Sciences, Khon Kaen University, Khon Kaen 40002, Thailand; 6 Department of Clinical Microscopy, Faculty of Associated Medical Sciences, Khon Kaen University, Khon Kaen 40002, Thailand; 7 Department of Clinical Immunology, Faculty of Associated Medical Sciences, Khon Kaen University, Khon Kaen 40002, Thailand; 8 Laboratory of Pharmacology, Chulabhorn Research Institute (CRI), Vibhavadee-Rangsit Highway, Laksi, Bangkok 10210, Thailand; 9 Department of Physiology, Faculty of Medicine, Khon Kaen University, Khon Kaen 40002, Thailand

**Keywords:** blood pressure, oxidative stress, malondialdehyde, sesamin, sesamolin, tocopherol

## Abstract

**Background:**

It has been known that hypertension is an independent risk factor for cardiovascular disease (CVD). CVD is the major cause of morbidity and mortality in developed and developing countries. Elevation of blood pressure (BP) increases the adverse effect for cardiovascular outcomes. Prevention of increased BP plays a crucial role in a reduction of those outcomes, leading to a decrease in mortality. Therefore, the purpose of this study was to investigate the effects of dietary black sesame meal on BP and oxidative stress in individuals with prehypertension.

**Methods:**

Twenty-two women and eight men (aged 49.8 ± 6.6 years) with prehypertension were randomly divided into two groups, 15 subjects per group. They ingested 2.52 g black sesame meal capsules or placebo capsules each day for 4 weeks. Blood samples were obtained after overnight fasting for measurement of plasma lipid, malondialdehyde (MDA) and vitamin E levels. Anthropometry, body composition and BP were measured before and after 4-week administration of black sesame meal or a placebo.

**Results:**

The results showed that 4-week administration of black sesame meal significantly decreased systolic BP (129.3 ± 6.8 vs. 121.0 ± 9.0 mmHg, *P *< 0.05) and MDA level (1.8 ± 0.6 vs. 1.2 ± 0.6 μmol/L, *P *< 0.05), and increased vitamin E level (29.4 ± 6.0 vs. 38.2 ± 7.8 μmol/L, *P *< 0.01). In the black sesame meal group, the change in SBP tended to be positively related to the change in MDA (*R = 0.50, P *= 0.05), while the change in DBP was negatively related to the change in vitamin E (*R = -0.55, P *< 0.05). There were no correlations between changes in BP and oxidative stress in the control group.

**Conclusions:**

These results suggest the possible antihypertensive effects of black sesame meal on improving antioxidant status and decreasing oxidant stress. These data may imply a beneficial effect of black sesame meal on prevention of CVD.

## Background

Hypertension is an important risk factor for cardiovascular disease (CVD) in developing countries. Elevation of blood pressure (BP) is a risk factor for adverse cardiovascular outcomes, including stroke, myocardial infarction, renal failure and death [1]. Prevention of increased BP therefore plays a crucial role in a reduction of those outcomes. Impaired balance between relaxing and contracting factors in the endothelium of blood vessels is an important pathogenic mechanism of hypertension. Increased pro-oxidant and decreased antioxidant activities have been shown to be some of the mechanisms of the pathogenesis of hypertension [2].

It has been reported that sesame seeds can improve oxidative stress due to actions of their contents of vitamin E and lignans including sesamin, sesamolin and sesamol [3-7]. Thus, sesame is likely to have potential health benefits in relation to CVD by its antihypertensive effects [8-11]. Although many previous studies have shown different effects of vitamin E on BP [12-14], these studies investigated the effect of supplementation of either alpha-tocopherol alone or mixed with gamma-tocopherol on BP in diabetic [12,13] or hypertensive [14] patients who took antihypertensive drugs. Interactions between alpha-tocopherol and the drug may diminish the antihypertensive effect on BP [15]. Moreover, the dose of vitamin E in those previous studies may have been so high that it caused increased or unchanged effects on BP.

Interestingly, the antihypertensive effect of black sesame (*Sesamum indicum *Linn.) meal, a product of sesame oil manufacturing, does not seem to have been previously investigated. Positive results may provide additional value to the manufactured product. Since we wanted to examine preventive effects, we performed the research on pre-hypertensive [16] healthy individuals who did not take any medicine; hence the vitamin E could exercise its antioxidant activity without interference with any antihypertensive drug. Moreover, black sesame meal contains gamma-tocopherol which was reported to be lower in patients with coronary heart disease than control subjects [17]. Taken together with the safety dose of vitamin E in black sesame meal in this study [18], this may reveal an anti-hypertensive effect of black sesame meal.

Based on knowledge of the effects of sesame seed and lignans on oxidative stress, which is one of mechanisms of the pathogenesis of hypertension [2], it was hypothesized that black sesame meal may have an antihypertensive effect in pre-hypertensive humans via improving antioxidant status and decreasing oxidant stress. Therefore, the aim of this study was to investigate the effects of 4-week administration of black sesame meal on BP and oxidative stress in pre-hypertensive humans.

## Methods

### Study design

A double-blind, placebo-controlled investigation was undertaken. Subjects were divided into two groups, with 11 men and 4 women in each group, matched by age, BMI and BP: a black sesame meal group (SG) (N = 15, aged 49.3 ± 7.7 years), and a placebo group (PG) (N = 15, aged 50.3 ± 5.6 years). Subjects and investigators were blinded as to the composition of the black sesame meal and placebo capsules. After being screened by physical and blood examination, all subjects were asked to complete health questionnaires in order to provide health information including cardiovascular risk factors. Subjects were seen on two visits: before and after 4-week administration of either black sesame meal capsules or placebo capsules. During the 4 weeks, subjects were asked to take 6 capsules each time, three times a day, with water after a meal. They were asked to avoid vitamins and other dietary supplements during the administration period. Subjects were instructed not to change their diet and exercise routine throughout the trial. At both visits, after a 12-h fasting period, blood samples were collected to measure blood parameters. Anthropometry (height, weight, and waist and hip circumferences), body composition and BP were also measured.

### Subjects

Thirty middle-aged subjects (22 men and 8 women) were recruited from the general population, supported by an annual health checkup program performed at the Faculty of Associated Medical Sciences, Khon Kaen University, during 2008-2009. Subjects had no other diseases except prehypertension as indicated either by systolic blood pressure (SBP) from 120 to 139 mmHg or diastolic BP (DBP) from 80 to 89 mmHg [16]. The patients were not currently taking any medication or dietary supplementation that affected BP. This study was conducted according to the guidelines laid down in the Declaration of Helsinki, and all procedures involving humans were approved by the Ethics Committee of Khon Kaen University (HE 510254). Written and verbal informed consent was obtained from all subjects; verbal consent was witnessed and formally recorded.

### Power calculation

A change in SBP after the ingestion of black sesame meal was used to calculate the sample size of this study [9]. The magnitude of the change in SBP was 5 mmHg for the power calculation. It was decided to require 90% power at a significance level of 0.05. Thus, having at least 15 subjects was required to finish this study.

### Preparation of black sesame meal

The black sesame meal was prepared at the Faculty of Pharmaceutical Sciences, Khon Kaen University, Thailand. The sesame seeds were roasted before being pressed. The remaining sesame meal was grounded into powder and mixed with an adsorbent. This process is the same as that used for commercial preparation. Each capsule of black sesame meal was formulated to contain 0.42 g of black sesame meal. The contents (mean ± SD) of carbohydrate, protein and fiber of the black sesame meal were 46.37%, 21.57% and 14.12%, respectively. Moreover, sesamin and sesamolin contents were 1.172 ± 0.002 mg/g DW and 0.605 ± 0.003 mg/g DW, respectively [19]. Total tocopherol was 105.41 ± 2.49 μg/g DW, mostly consisting of gamma-tocopherol (102.78 ± 2.46 μg/g DW); alpha-tocopherol was not detectable [19]. The placebo capsule contained the same contents, but without any black sesame meal. Both capsules were the same shape and color.

### Measurement of BP

BP (mmHg) and heart rate (HR,/min) were measured in the morning, after a 20-min rest, using an automatic sphygmomanometer (Accurtorr 1A; Datascope, Japan) on the right upper arm while the subject was in a sitting position. Average BP and HR were calculated from three measurements after an almost stable BP seemed to have been reached. SBP and DBP were used to calculate mean arterial pressure (MAP) by the formula: MAP = DBP + 1/3 (SBP - DBP).

### Measurement of malondialdehyde (MDA)

A plasma lipid peroxidation marker, MDA, was estimated using thiobarbituric acid, as previously described [20]. In brief, 150 μl of plasma was reacted with 125 μl 10% trichloroacetic acid, 125 μl 5 mM ethylenediaminetetraacetic acid, 125 μl 8% sodium dodecyl sulfate, and 10 μl 0.5 μg/ml butylated hydroxytoluene. After vortexing vigorously for 30 s, the mixture was incubated for 10 min at room temperature. Then 500 μl 0.6% thiobarbituric acid was added, and the mixture was heated at 95°C for 30 min. After cooling to room temperature, the mixture was centrifuged at 10,000 rpm for 10 min. The absorbance of the supernatant was measured at 532 nm using a spectrophotometer (Genesys 20, SN: 35 gk 130009; Thermo Fisher Scientific, USA). A standard curve was generated with appropriate concentrations of 1,1,3,3-tetraethoxypropane standard (0.3-10 μmol/L); the plasma MDA concentration was expressed as μmol/L of plasma.

### Measurement of vitamin E

Serum vitamin E was determined using high performance liquid chromatography (HPLC), according to the method of Thurnham [21], by injection into a reversed-phase C-18 Spherisorb ODS2 column (diameter 5 μm, 4.6 × 100 mm; Waters, USA). The mobile phase consisted of methanol/acetonitrile/dichloromethane at a ratio of 4:4:1 with a flow rate of 1 mL/min. A wavelength UV-visible detector (model 2847, Waters) was set at a wavelength of 292 nm for the detection of vitamin E. Quantification was based on peak-height measurement, using the Clarity 2.2.0.67 software program, version C22 (DataApex, Czech Republic).

### Statistical analysis

All statistics were generated using Stata statistical software, version 10 (StataCorp, USA). Data were expressed as means ± SD. Changes in BP, MDA and vitamin E levels between groups were compared by analysis of covariance (ANCOVA), with adjustment for baseline values. The variables under study before and after the administration within groups were compared by Student's paired *t*-test. The relationships between changes in oxidative stress variables and BP were analyzed by Pearson's correlation. Statistical analyses were two-sided. If the statistical probability (*P *-value) was less than 0.05, the differences were considered to be statistically significant.

## Results

Baseline characteristics of subjects are shown in Table 1. All subjects were pre-hypertensive individuals. No significant differences in age, anthropometry, body composition, and levels of MDA and vitamin E, SBP, DBP, MAP and HR were found between SG and PG groups.

**Table 1:** Characteristics and baseline outcome of the subjects at baseline

	PG (n = 15)	SG (n = 15)	*P*-value
Age (yr)	50.3 ± 5.6 (39-58)	49.3 ± 7.7 (38-59)	0.70
Height (cm)	161.4 ± 5.4 (154-171)	159.5 ± 7.5 (150-173)	0.45
BW (kg)	66.9 ± 8.7 (53.9-78.5)	68.1 ± 12.1 (57.5-92.7)	0.71
BMI (kg/m^2^)	25.6 ± 2.4 (21.8-29.1)	26.6 ± 3.2 (22.7-34.3)	0.29
%BF	31.5 ± 4.6 (24.7-37.6)	31.6 ± 3.4 (26.7-37.2)	0.92
FM (kg)	21.3 ± 5.1 (13.3-28.5)	21.7 ± 5.2 (15.9-32.7)	0.81
FFM (kg)	45.6 ± 4.7 (37.6-52.9)	46.4 ± 7.6 (37.7-60.9)	0.69
W (cm)	84.6 ± 6.9 (72-94)	85.8 ± 9.6 (74.5-107)	0.66
H (cm)	95.3 ± 4.9 (86-101.5)	96.9 ± 5.4 (88-108)	0.35
W/H ratio	0.89 ± 0.1 (0.77-0.94)	0.88 ± 0.1 (0.79-1.02)	0.75
MDA (μmol/L)	1.9 ± 0.62 (0.86-2.78)	1.8 ± 0.6 (1.19-2.81)	0.67
Vitamin E (μmol/L)	26.5 ± 5.5 (17.2-34.4)	29.4 ± 6.0 (19.1-45.8)	0.18
SBP (mmHg)	130.4 ± 4.8 (127-138)	129.3 ± 6.8 (121-137)	0.43
DBP (mmHg)	80.6 ± 7.7 (72-99)	77.0 ± 7.4 (65-87)	0.63
MAP (mmHg)	97.2 ± 4.7 (90.3-108.7)	94.4 ± 6.1 (84.7-103.7)	0.51
HR (/min)	66.8 ± 7.4 (59-85)	68.7 ± 9.1 (53-93)	0.61

Values are expressed as means ± SD (minimum-maximum).

PG, placebo group; SG, black sesame meal group; BW, body weight; BMI, body mass index; %BF, percentage of body fat; FM, fat mass; FFM, fat-free mass; W, waist circumference; H, hip circumference; W/H ratio, waist and hip circumference ratio; MDA, malondialdehyde; SBP, systolic blood pressure; DBP, diastolic blood pressure; MAP, mean arterial pressure; HR, heart rate

**Figure 1:**
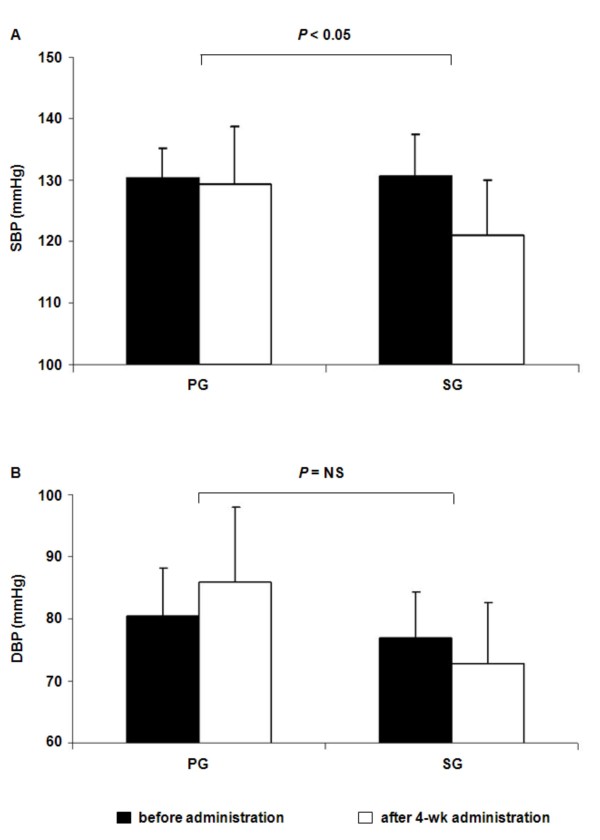
**Average levels of SBP (A) and DBP (B) before and after 4-week administration of black sesame meal and a placebo in subjects with prehypertension**. Values are expressed as means ± SD. SBP, systolic blood pressure; DBP, diastolic blood pressure; PG, placebo group; SG, black sesame meal group.

In the SG group, after 4 weeks of administration of black sesame meal, SBP was significantly decreased when compared with before administration (121.0 ± 9.0 vs. 129.3 ± 6.8 mmHg, *P *< 0.05) (Figure 1A). Meanwhile, in the PG group, SBP was slightly decreased after the placebo administration when compared with before administration values (130.4 ± 4.8 vs. 130.6 ± 9.5 mmHg, *P *= 0.52). The decrease in SBP in the SG group was significantly greater than that in the PG group (P < 0.05) with adjustment for baseline values. There were no apparent side effects induced by black sesame meal throughout the study.

In the SG group, after 4 weeks of administration of black sesame meal, DBP was decreased when compared with before administration (72.8 ± 9.8 vs. 77.0 ± 7.4 mmHg, *P *= 0.20) (Figure 1B). Meanwhile, in the PG group (85.9 ± 12.1 vs. 80.6 ± 7.7 mmHg, *P *= 0.14), where the level was higher than before administration. However, the changes in DBP between groups with adjustment for baseline values were not significantly different.

Moreover, there was no significant difference in changes of average HR with adjustment for baseline values in both PG and SG subjects (66.8 ± 7.4 and 68.7 ± 9.1/min before and 71.2 ± 11.1 and 71.4 ± 6.4/min after 4-week administration of black sesame meal and placebo, *P *= 0.76).

**Figure 2:**
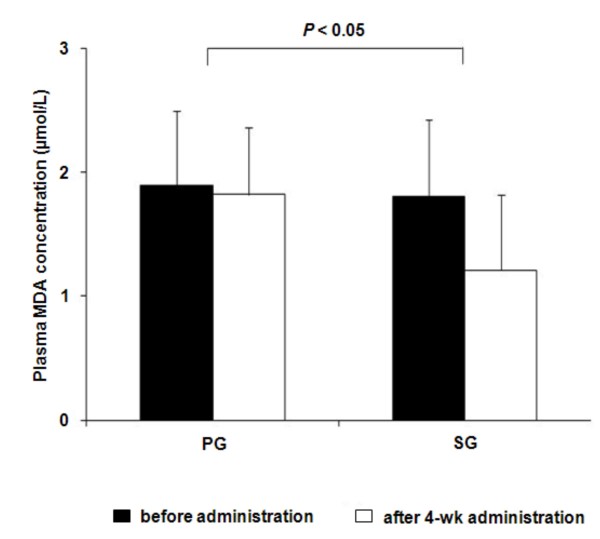
**Plasma MDA concentrations before and after 4-week administration of black sesame meal and a placebo in subjects with prehypertension**. Values are expressed as means ± SD. PG, placebo group; SG, black sesame meal group; MDA, malondialdehyde.

Plasma MDA concentrations significantly decreased after the 4-week administration of black sesame meal when compared with before administration concentrations (1.2 ± 0.6 vs. 1.8 ± 0.6 μmol/L, *P *< 0.05). There were no significant differences in MDA concentrations, however, before and after 4-week administration of a placebo (1.8 ± 0.5 vs. 1.9 ± 0.6 μmol/L, *P *= NS). When compared with the PG subjects, the SG subjects had significantly lower plasma MDA concentrations after the 4-week administration with adjustment for baseline values (1.8 ± 0.5 (PG), 1.2 ± 0.6 (SG) μmol/L, *P *< 0.05) (Figure 2).

**Figure 3:**
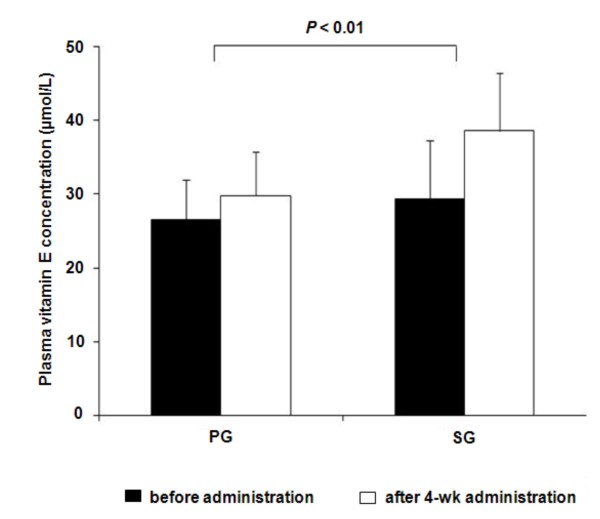
**Plasma vitamin E concentrations before and after 4-week administration of black sesame meal and a placebo in subjects with prehypertension**. Values are expressed as means ± SD. PG, placebo group; SG, black sesame meal group.

As shown in Figure 3, plasma vitamin E (total tocopherol) concentrations after 4-week administration of black sesame meal were significantly increased when compared with before administration concentrations (38.2 ± 7.8 vs. 29.4 ± 6.0 μmol/L, *P *< 0.01). There were no significant differences in vitamin E concentrations before and after 4-week administration of the placebo (29.8 ± 6.0 vs. 26.5 ± 5.5 μmol/L, *P *= NS). When compared with the PG group, the SG group had significantly greater plasma vitamin E concentrations after 4-week of administration with adjustment for baseline values (29.8 ± 6.0 (PG), 38.2 ± 7.8 (SG) μmol/L, *P *< 0.01).

Only in the black sesame meal group did the change in SBP tend to be positively related to the change in MDA concentration (*R = 0.50, P *= 0.05), while the change in DBP was negatively related to the change in vitamin E concentration (*R = 0.55, P *< 0.05). There were no significant correlations between changes in BP and oxidative stress indicators in the placebo group.

## Discussion

This study appears to be the first to reveal the possible antihypertensive effects of black sesame meal in participants with prehypertension. The results demonstrated that after matching for age, BMI and BP, 4-week daily administration of 2.52 g black sesame meal caused a significant reduction in SBP, by an average of 8.2 mmHg.

Interestingly, the INTERSALT study revealed that a reduction of 2 to 3 mmHg of SBP was associated with a 4% decrease in mortality from CVD in the USA and UK [22] and a 6.4% decrease in mortality from cerebral vascular disease in Japan [1]. Based on these longer term studies, if the present reduction in BP with sesame meal (8.2 mmHg) was sustained in the long term, this could reduce the risk of CVD and stroke by 16.4% and 26.2% respectively. This study shows that the ingestion of black sesame meal may have a potential effect on reduction in mortality from CVD and stroke.

Although no previous study has directly investigated the effect of black sesame meal on BP, many studies have reported the potential antihypertensive effect of sesame's contents (i.e. lignan and vitamin E) in humans and rats [8-11,23]. However, this is inconsistent with many other previous studies which reported different results [12-14]. Some previous studies have reported that supplementation of either alpha-tocopherol alone or mixed with gamma-tocopherol increased [12] or did not change [13] BP in diabetic or treated hypertensive patients [14]. This may be attributed to an interference of alpha-tocopherol and the antihypertensive drug taken by some subjects in previous studies; whereas subjects in this study were healthy and did not take any antihypertensive drug. Moreover, the dose of vitamin E in those previous studies may have been so high that it caused increased or unchanged effects on BP.

A possible mechanism responsible for the antihypertensive effect of black sesame meal in this study may be an improved balance between relaxing and contracting factors in the endothelium of blood vessels. In this study, black sesame meal may have enhanced the relaxing factor, resulting in improved endothelium-dependent vasorelaxation in the pre-hypertensive participants relating to oxidative stress. The antihypertensive effect of sesamin, in mildly hypertensive humans was supported by a previous study [9]. The authors reported that the ingestion of 60 g of sesamin per day for 4 weeks decreased SBP by an average of 3.5 mmHg, and DBP by 1.9 mmHg [9]. Moreover, the increased plasma vitamin E in this study may be due to the increased vitamin E or the inhibition of catabolism of vitamin E. In addition, bioavailability of vitamin E may be increased by interactions between sesame lignan and tocopherols [24]. The accumulation of vitamin E acts by detoxifying the hydroxyl and proxy radicals, leading to reduced lipid peroxidation, or by reducing excess tissue aldehydes [11]. This is supported by many previous studies, in which supplementation of either vitamin E or sesamin and sesamolin demonstrated that these antioxidants inhibit lipid peroxidation [3-7,25]. The antioxidant effect is likely to contribute to the decreased endothelial dysfunction from free radicals [26], resulting in an increase in the vasorelaxing factor, nitric oxide (NO) [27].

However, a mechanism that is not relevant to oxidative stress cannot be ruled out. This was supported by a study by Ward et al. (2004) which failed to show a link between oxidative stress and BP [28]. It was shown that gamma-tocopherol supplementation increased protein expression of nitric oxide synthase [29], which stimulates vasorelaxation. Moreover, the potential effect of vitamin E on membrane fluidity is likely to be another mechanism of antihypertension. In vitro study, vitamin E was shown to preserve endothelial cell (EC) migration in oxidized low-density lipoprotein cells (oxLDL) and restore the endothelial monolayer after injury by inhibiting changes in membrane integrity caused by oxLDL [30]. A previous study in rats demonstrated that antihypertensive rats had lower BP than normotensive rats due to reduced membrane fluidity after ingestion of vitamin E 3 d/week for 3 weeks [31]. These non-antioxidant properties of vitamin E could be important in the prevention of atherosclerosis, resulting in a reduction in BP in humans. Sesamin supplementation also induced NO and inhibited endothelin-1 production by EC [32]. These findings indicate an improved ability of the endothelium to relax. In addition, a recent study has shown that sesamin inhibited some CYP450 enzymes and the production of 20-hydroxyeicosatetraenoic acid (20-HETE), which might influence BP independently of any effects on oxidative stress [33]. Moreover, sesamin increased Ca^2+ ^antagonistic vasorelaxing activity [34]. It should be emphasized in this discussion that these hypotheses are only on the basis of the decrease in MDA and increased vitamin E, because other possible mechanisms were not measured.

A limitation of this study is a lack of data on endothelium-dependent vasorelaxation determined by NO and other antioxidants such as vitamin C. Moreover, the potential effect of vitamin E on membrane fluidity and the ability of sesame lignans to inhibit 20-HETE synthesis in human renal and liver microsomes were not investigated by this study. Thus, further investigation of these variables explaining the mechanisms of black sesame meal on prevention of hypertension is needed. Importantly, having no discernible side effects from the ingestion of black sesame meal throughout this study implies that it may be safe as a nutritional supplement for health promotion.

## Conclusions

This study suggests a beneficial effect of dietary black sesame meal on a reduction in blood pressure in pre-hypertensive humans. It is likely that the antihypertensive effect is due to decreased oxidative stress. Taken together with the absence of side effects and inexpensive preparation, the regular ingestion of dietary black sesame meal may be beneficial for CVD prevention in individuals with prehypertension, or even those with hypertension. A future study that investigates this advantageous effect is suggested.

## Competing interests

The authors are applying for a patent on black sesame meal.

## Authors' contributions

JW participated in the design of the study and carried out the plasma MDA and vitamin E assay, physiological measurements and statistical analysis. NW prepared the black sesame meal. PB carried out the vitamin E assay. SS performed the medical cover. NL conceived the study, participated in its design and coordination. NR analyzed vitamin E and lignans in black sesame meal. JW and NL drafted the manuscript. NL is a member of Exercise and Sport Sciences Development and Research Group. NW, CS, NS, NK, SS and YT are members of Cardiovascular Research Group. All authors have read and approved the final manuscript.

## References

[B1] Japanese Society of Hypertension Japanese Society of Hypertension guidelines for the management of hypertension (JSH 2004) Hypertens Res 2006 29 Suppl S1 S105 10.1291/hypres.29.S1 17366911

[B2] Dhalla NS Temsah RM Netticadan T Role of oxidative stress in cardiovascular diseases J Hypertens 2000 18 655 673 10.1097/00004872-200018060-00002 10872549

[B3] Cooney RV Custer LJ Okinaka L Franke AA Effects of dietary sesame seeds on plasma tocopherol levels Nutr Cancer 2001 39 66 71 10.1207/S15327914nc391_9 11588904

[B4] Ikeda S Kagaya M Kobayashi K Tohyama T Kiso Y Higuchi N Yamashita K Dietary sesame lignans decrease lipid peroxidation in rats fed docosahexaenoic acid J Nutr Sci Vitaminol (Tokyo) 2003 49 270 276 10.3177/jnsv.49.270 14598914

[B5] Kang MH Naito M Tsujihara N Osawa T Sesamolin inhibits lipid peroxidation in rat liver and kidney J Nutr 1998 128 1018 1022 9614163 10.1093/jn/128.6.1018

[B6] Nakai M Harada M Nakahara K Akimoto K Shibata H Miki W Kiso Y Novel antioxidative metabolites in rat liver with ingested sesamin J Agric Food Chem 2003 51 1666 1670 10.1021/jf0258961 12617602

[B7] Yamashita K Iizuka Y Imai T Namiki M Sesame seed and its lignans produce marked enhancement of vitamin E activity in rats fed a low alpha-tocopherol diet Lipids 1995 30 1019 1028 10.1007/BF02536287 8569430

[B8] Boshtam M Rafiei M Sadeghi K Sarraf-Zadegan N Vitamin E can reduce blood pressure in mild hypertensives Int J Vitam Nutr Res 2002 72 309 314 10.1024/0300-9831.72.5.309 12463106

[B9] Miyawaki T Aono H Toyoda-Ono Y Maeda H Kiso Y Moriyama K Antihypertensive effects of sesamin in humans J Nutr Sci Vitaminol (Tokyo) 2009 55 87 91 10.3177/jnsv.55.87 19352068

[B10] Sankar D Rao MR Sambandam G Pugalendi KV Effect of sesame oil on diuretics or β-blockers in the modulation of blood pressure, anthropometry, lipid profile, and redox status Yale J Biol Med 2006 79 19 26 17876372 PMC1942178

[B11] Sankar D Sambandam G Rao MR Pugalendi KV Modulation of blood pressure, lipid profiles and redox status in hypertensive patients taking different edible oils Clin Chim Acta 2005 355 97 104 10.1016/j.cccn.2004.12.009 15820483

[B12] Ward NC Wu JH Clarke MW Puddey IB Burke V Croft KD Hodgson JM The effect of vitamin E on blood pressure in individuals with type 2 diabetes: a randomized, double-blind, placebo-controlled trial J Hypertens 2007 25 227 234 10.1097/01.hjh.0000254373.96111.43 17143195

[B13] Economides PA Khaodhiar L Caselli A Caballero AE Keenan H Bursell SE King GL Johnstone MT Horton RS Veves A The effect of vitamin E on endothelial function of micro- and macrocirculation and left ventricular function in type 1 and type 2 diabetic patients Diabetes 2005 54 204 211 10.2337/diabetes.54.1.204 15616030

[B14] Palumbo G Avanzini F Alli C Roncaglioni MC Ronchi E Cristofari M Capra A Rossi S Nosotti L Costantini C Cavalera C Effects of vitamin E on clinic and ambulatory blood pressure in treated hypertensive patients. Collaborative Group of the Primary Prevention Project (PPP) - Hypertension study Am J Hypertens 2000 5 Pt 1 564 567 10.1016/s0895-7061(00)00244-2 10826412

[B15] Brigelius-Flohé R Vitamin E and drug metabolism Biochem Biophys Res Commun 2003 305 737 740 10.1016/S0006-291X(03)00811-8 12763054

[B16] Chobanian AV Bakris GL Black HR Cushman WC Green LA Izzo JL Jr Jones DW Materson BJ Oparil S Wright JT Jr Roccella EJ The Seventh Report of the Joint National Committee on Prevention, Detection, Evaluation, and Treatment of High Blood Pressure: The JNC 7 Report JAMA 2003 289 2560 2571 10.1001/jama.289.19.2560 12748199

[B17] Kontush A Spranger T Reich A Baum K Beisiegel U Lipophilic antioxidants in blood plasma as markers of atherosclerosis: the role of alpha-carotene and gamma-tocopherol Atherosclerosis 1999 144 117 122 10381285 10.1016/s0021-9150(99)00044-1

[B18] Institute of Medicine, Food and Nutrition Board Dietary Reference Intakes for Vitamin C, Vitamin E, Selenium, and Carotenoids 2000 Washington, DC: National Academy Press 25077263

[B19] Rangkadilok N Pholphana N Mahidol C Wongyai W Saengsooksree K Nookabkaew S Satayavivad J Variation of sesamin, sesamolin and tocopherols in sesame (*Sesamum indicum *L.) seeds and oil products in Thailand Food Chemistry 2010 122 724 730 10.1016/j.foodchem.2010.03.044

[B20] Draper HH Squires EJ Mahmoodi H Wu J Agarwal S Hadley M A comparative evaluation of thiobarbituric acid methods for the determination of malondialdehyde in biological materials Free Radic Biol Med 1993 15 353 363 10.1016/0891-5849(93)90035-S 8225017

[B21] Thurnham DI Smith E Flora PS Concurrent liquid-chromatographic assay of retinol, alpha-tocopherol, beta-carotene, alpha-carotene, lycopene, and beta-cryptoxanthin in plasma, with tocopherol acetate as internal standard Clin Chem 1988 34 377 381 3342512

[B22] Stamler J Rose G Stamler R Elliott P Dyer A Marmot M INTERSALT study finding: public health and medical care implications Hypertension 1989 14 570 577 2807518 10.1161/01.hyp.14.5.570

[B23] Noguchi T Ikeda K Sasaki Y Yamamoto J Yamori Y Effects of vitamin E and sesamin on hypertension and cerebral thrombogenesis in stroke-prone spontaneously hypertensive rats Clin Exp Pharmacol Physiol 2004 31 Suppl 2 24 26 10.1111/j.1440-1681.2004.04103.x 15649279

[B24] Kamal-Eldin A Frank J Razdan A Tengblad S Basu S Vessby B Effects of dietary phenolic compounds on tocopherol, cholesterol, and fatty acids in rats Lipids 2000 35 427 435 10.1007/s11745-000-541-y 10858028

[B25] Ikeda S Abe C Uchida T Ichikawa T Horio F Yamashita K Dietary sesame seed and its lignan increase both ascorbic acid concentration in some tissues and urinary excretion by stimulating biosynthesis in rats J Nutr Sci Vitaminol (Tokyo) 2007 53 383 392 10.3177/jnsv.53.383 18079604

[B26] Ito H Torii M Suzuki T Comparative study on free radical injury in the endothelium of SHR and WKY aorta Clin Exp Pharmacol Physiol 1995 22 Suppl 2 157 159 9072336 10.1111/j.1440-1681.1995.tb02862.x

[B27] Thomas SR Chen K Keaney JF Jr Oxidative stress and endothelial nitric oxide bioactivity Antioxid Redox Signal 2003 5 181 194 10.1089/152308603764816541 12716478

[B28] Ward NC Hodgson JM Puddey IB Mori TA Beilin LJ Croft KD Oxidative stress in human hypertension: association with antihypertensive treatment, gender, nutrition, and lifestyle Free Radical Biol Med 2004 36 226 232 10.1016/j.freeradbiomed.2003.10.021 14744634

[B29] Li D Saldeen T Romeo F Mehta JL Relative effects of alpha- and gamma-tocopherol on low-density lipoprotein oxidation and superoxide dismutase and nitric oxide synthase activity and protein expression in rats J Cardiovasc Pharmacol Ther 1999 4 219 226 10.1177/107424849900400403 10684543

[B30] van Aalst JA Burmeister W Fox PL Graham LM Alpha-tocopherol preserves endothelial cell migration in the presence of cell-oxidized low-density lipoprotein by inhibiting changes in cell membrane fluidity J Vasc Surg 2004 39 229 237 10.1016/S0741-5214(03)01038-3 14718844

[B31] Pezeshk A Derick Dalhouse A Vitamin E, membrane fluidity, and blood pressure in hypertensive and normotensive rats Life Sci 2000 67 1881 1889 10.1016/S0024-3205(00)00775-X 11043610

[B32] Lee CC Chen PR Lin S Tsai SC Wang BW Chen WW Tsai CE Shyu KG Sesamin induces nitric oxide and decreases endothelin-1 production in HUVECs: possible implications for its antihypertensive effect J Hypertens 2004 22 2329 2338 10.1097/00004872-200412000-00015 15614027

[B33] Wu JH Hodgson JM Clarke MW Indrawan AP Barden AE Puddey IB Croft KD Inhibition of 20-hydroxyeicosatetraenoic acid synthesis using specific plant lignans: in vitro and human studies Hypertension 2009 54 1151 1158 10.1161/HYPERTENSIONAHA.109.139352 19786646

[B34] Nakano D Kwak CJ Fujii K Ikemura K Satake A Ohkita M Takaoka M Ono Y Nakai M Tomimori N Kiso Y Matsumura Y Sesamin metabolites induce an endothelial nitric oxide-dependent vasorelaxation through their antioxidative property-independent mechanisms: possible involvement of the metabolites in the antihypertensive effect of sesamin J Pharmacol Exp Ther 2006 318 328 335 10.1124/jpet.105.100149 16597711

